# 

**Published:** 1853-08

**Authors:** 


Vol. I. AUGUST, 1853. No. 1.

### IOWA

### MEDICAL JOURNAL

CONDUCTED BY THE FACULTY OF THE

**Medical Department of the Iowa University.**

PUBLISHED MONTHLY.

TERMS--TWO DOLLARS IN ADVANCE.

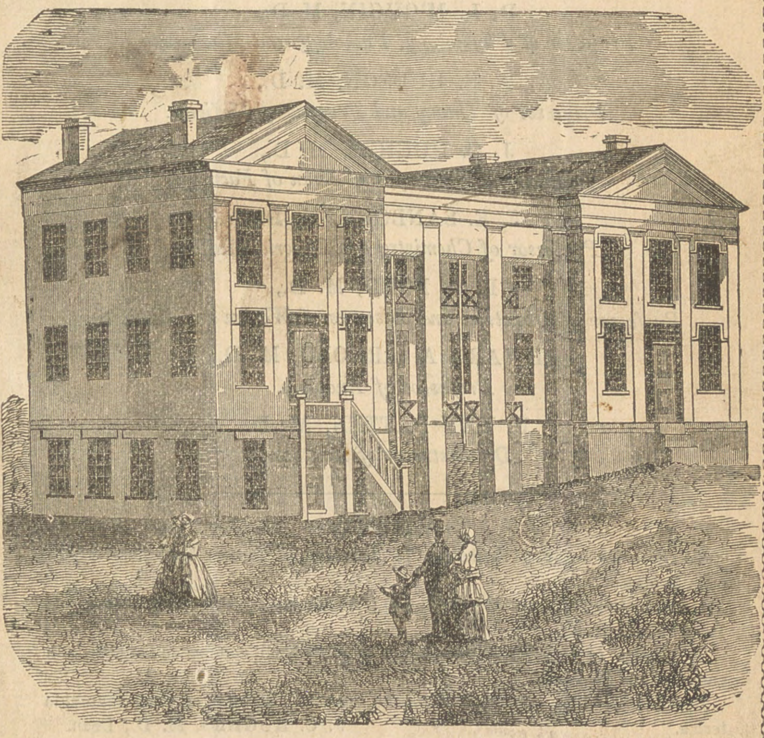

KEOKUK, IOWA:

Printed at the Whig Book and Job Office.

